# The Influence of Alloying Elements on the Hot Corrosion Behavior of Nickel-Based Superalloys

**DOI:** 10.3390/ma18091996

**Published:** 2025-04-28

**Authors:** Teodor-Adrian Badea, Mădălin Dombrovschi

**Affiliations:** Romanian Research and Development Institute for Gas Turbines COMOTI, 220D Iuliu Maniu Av., 061126 Bucharest, Romania; teodor.badea@comoti.ro

**Keywords:** hot corrosion, Inconel 625, Inconel 718, Nimonic 75, Udimet 710

## Abstract

Nickel-based superalloys are extensively used in high-temperature applications because of their exceptional oxidation and corrosion resistance. However, their performance in aggressive environments containing molten salts, such as Na_2_SO_4_ and V_2_O_5_, remains a critical challenge. This study investigated the hot corrosion behavior of Inconel 718, Udimet 710, Nimonic 75, and Inconel 625, focusing on the role of the alloying elements in the corrosion layers and degradation mechanisms. The superalloys were exposed to 50/50 wt.% Na_2_SO_4_–V_2_O_5_ at 900 °C for 8, 48, and 96 h, and their corrosion resistance was evaluated through weight gain measurements, scanning electron microscopy (SEM), and energy-dispersive X-ray spectroscopy (EDS). These results indicate that Mo is a key factor in accelerating degradation, with Inconel 625 exhibiting the highest weight gain owing to the formation of thermally unstable Mo-rich phases. Fe also negatively impacted the stability of the protective scale of Inconel 718, contributing to an increased corrosion rate. In contrast, Nimonic 75 exhibited the best resistance, forming more of the NiCr_2_O_4_ spinel phase through the reaction of Cr_2_O_3_ with NiO from the high Ni and Cr contents in the corrosive layers. These findings highlight the importance of alloy composition in optimizing corrosion resistance and suggest that using superalloys with lower Mo and Fe contents and higher Cr and Ni concentrations can significantly enhance the durability of superalloys in molten salt environments.

## 1. Introduction

Nickel-based superalloys are widely used in high-temperature environments, such as in power generation, aerospace, and marine industries, owing to their exceptional oxidation, mechanical, and corrosion resistance properties [1]. However, in highly aggressive environments containing molten salts, such as sodium sulfate (Na_2_SO_4_) and vanadium pentoxide (V_2_O_5_), these alloys are susceptible to hot corrosion, which can significantly degrade their structural integrity and lifespan. Hot corrosion emerged as a significant issue in the 1940s, as evidenced by the deterioration of steam-plant boiler tubes. This phenomenon remained under close scrutiny until the late 1960s, when it became a critical concern owing to the corrosive damage inflicted on helicopter engine components during the Vietnam War, particularly affecting aircraft operating in or near maritime environments [2,3].

Hot corrosion poses a significant threat to Ni-based superalloys when exposed to molten-salt-containing environments. These materials experience enhanced oxidation and diminished corrosion resistance upon contact with Na_2_SO_4_ deposits at high temperatures. Interactions with molten salts can lead to the development of intricate corrosion scales and internal sulfidation, negatively affecting the performance of alloys [4].

This is particularly critical in gas turbines, jet engines, and industrial boilers, where fuel impurities introduce corrosive deposits that accelerate material deterioration and failure [5]. Among the most widely used nickel-based superalloys in these industries, Inconel 718, Udimet 710, Nimonic 75, and Inconel 625 exhibit varying degrees of hot corrosion resistance, which is largely influenced by their chemical compositions [6].

Inconel 718, a precipitation-hardened superalloy, is widely used because of its high oxidation and corrosion resistance at temperatures of 600–700 °C and beyond. However, in Na_2_SO_4_ and V_2_O_5_ environments, molten salts degrade the chromium oxide (Cr_2_O_3_) protective scale, leading to corrosion, sulfidation, and phase destabilization [7]. A recent study analyzed Al and Cr oxides and showed that aluminum oxide (Al_2_O_3_) acts as a diffusion barrier, preventing oxygen ingress and Cr depletion, thereby outperforming Cr_2_O_3_ in terms of long-term corrosion resistance [8]. In addition, a study showed that to improve hot corrosion resistance, alloy modifications through reduced Mo/W content are being explored, although challenges remain in terms of long-term adhesion and thermal cycling stability [9]. Additionally, research on laser-repaired Inconel 718 under hot corrosion (Na_2_SO_4_ + NaCl + NaNO_3_ at 650 °C) indicates that Cr depletion, Ni sulfide formation, and corrosion initially strengthen the alloy but eventually lead to δ-phase formation, reducing mechanical performance. Thermal effects dominated the degradation, emphasizing the need for improved repair strategies and protective coatings [10]. In addition, when exposed to molten salt environments, superalloys containing Fe typically develop iron oxides, such as Fe_2_O_3_, and sulfides, which lack protective properties. This formation contributes to the decreased resistance of the alloys to hot corrosion [11].

Udimet 710 is a high-temperature nickel-based superalloy with γ′ strengthening, offering excellent oxidation and creep resistance up to 750–800 °C, but is susceptible to hot corrosion in Na_2_SO_4_ and V_2_O_5_ environments [12]. However, the molybdenum (Mo) and tungsten (W) contents of the alloy can contribute to low-melting-point phases, worsening its corrosion behavior [13]. In addition, the ratio of Al to Ti in superalloys is critical for developing a dense and protective NiTiO_3_ layer, which can substantially enhance the resistance to hot corrosion [14].

Notably, the hot corrosion resistance of an alloy is significantly influenced by its composition. A study on Fe-Mn-Al-C alloys demonstrated that increasing the Al content enhanced the corrosion resistance at 850 °C. In contrast, although the addition of Cr decreased the metal loss, it did not substantially improve the overall hot corrosion resistance. These findings suggest that the specific elements used in alloying UDIMET 710 have a direct impact on its hot corrosion resistance [15].

Nimonic 75 is a Ni-Cr alloy with moderate strength, oxidation resistance, and stability up to 750 °C; however, it is prone to hot corrosion in Na_2_SO_4_ and V_2_O_5_ environments owing to Cr_2_O_3_ scale degradation. In the absence of γ′ strengthening, it is more susceptible to creep [16].

Inconel 625, a solid-solution-strengthened superalloy, offers high oxidation stability and corrosion resistance up to 980 °C. However, the Cr_2_O_3_ scales degrade in molten salt environments, causing sulfidation and phase instability. While Mo enhances the mechanical properties, it can also promote low-melting-point phase formation, worsening hot corrosion [17].

The resistance of these superalloys to hot corrosion is dictated by their alloying elements, which influence the formation and stability of protective oxide layers. Cr and Al are stable oxides, whereas Mo and W can contribute to low-melting-point phases that accelerate degradation [14]. Additionally, recent studies on elemental segregation in Inconel 718 using microbeam X-ray fluorescence (µ-XRF) spectroscopy have provided new insights into the role of elemental distribution in the high-temperature performance. The findings revealed severe segregation of niobium (Nb) and titanium (Ti), particularly in Nb-rich precipitate zones, which can affect corrosion resistance and mechanical stability. This study underscores the importance of controlling elemental segregation to optimize the hot corrosion resistance of nickel-based superalloys [18].

Understanding how these elements interact with Na_2_SO_4_ and V_2_O_5_ at high temperatures is crucial for selecting the appropriate superalloy for extreme operating environments. The current study aims to investigate the impact of alloying elements on the hot corrosion behavior of Inconel 718, Udimet 710, Nimonic 75, and Inconel 625 when exposed to Na_2_SO_4_ and V_2_O_5_. This study elucidates the mechanisms governing hot corrosion and supports the identification of key alloying elements for improving the resistance. Therefore, the insights gained from this study will contribute to the selection of more durable superalloys for high-temperature applications, ultimately enhancing the reliability and longevity of critical components in extreme environments.

## 2. Materials and Methods

**Figure 1:**
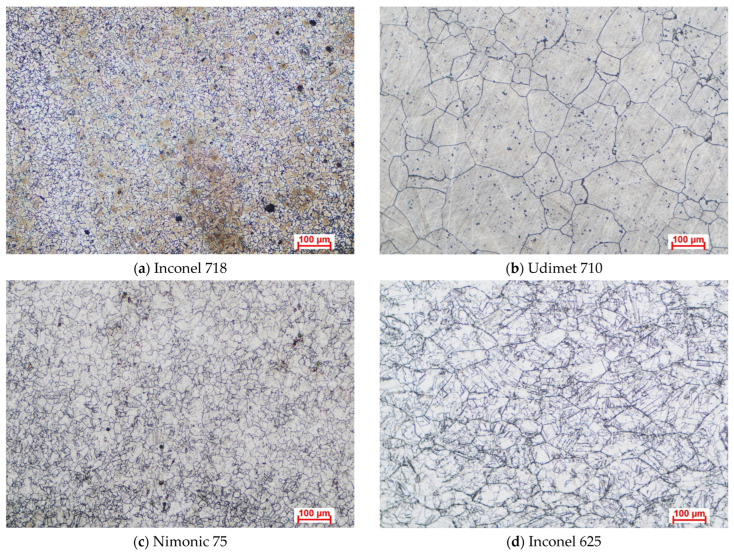
Microstructure of materials utilized.

In this study, Inconel 718, Udimet 710, Nimonic 75, and Inconel 625 samples were used for the experiments. The Inconel 718 and Udimet 710 samples were supplied in the solution-annealed and aged condition, having undergone heat treatment to dissolve segregated phases and promote the formation of γ′ and γ″ strengthening precipitates. Nimonic 75 was provided in the annealed condition, ensuring a homogeneous solid solution. Inconel 625 was delivered in the solution-annealed state, where alloying elements were fully dissolved into the austenitic matrix to enhance corrosion resistance and prepare the material for high-temperature applications. The samples used in this study were purchased from Bibus Metals, located in Păulești, Romania, a certified supplier of high-performance alloys for industrial and research applications. The mass of each sample was measured using a Cole-Parmer Ohaus PX224 Pioneer analytical balance (Antylia Scientific, Vernon Hills, IL, USA) with an accuracy of 10^−4^ g. The samples were cut from sheets of each material into dimensions of approximately 15 mm × 15 mm × 3 mm. Surface preparation was performed by grinding with SiC abrasive paper up to 1200 grit granulation, followed by cleaning with deionized water, ultrasonic degreasing in acetone, and air-drying on filter paper. The cross-sectional microstructural characteristics of the samples are depicted in Figure 1 as optical microscopy (OM) images obtained using an Axio Vert (NIS Elements 5.0). An A1 MAT optical microscope (Carl Zeiss Microscopy GmbH, Jena, Germany) was used for the analysis. The polished sample surfaces were etched by immersion in aqua regia for 10–15 s to reveal their microstructural features.

The chemical compositions of the raw materials were analyzed using optical emission spectrometry (OES) (WAS PMI-MASTER PLUS, Oxford Instruments, Abingdon, UK), and the results are listed in Table 1.

In the hot corrosion tests, a powder mixture of 50 wt.% Na_2_SO_4_ and 50 wt.% V_2_O_5_ was uniformly applied to the surfaces of the samples. To ensure accurate and consistent application, each specimen was weighed both before and after the powder deposition, allowing for precise calculation of the applied salt quantity and verification of the target area density of ~5 mg/cm^2^. The mixture was distributed using a fine brush to promote even surface coverage and minimize agglomeration or loss. This approach ensured uniform exposure conditions across all samples, improving the reliability and reproducibility of the corrosion results.

The processed samples were placed in alumina crucibles and subjected to heat treatment in a Nabertherm LH 30/14 furnace (Lilienthal, Germany). The heating rate was set to 250 °C/h, and the temperature was increased from room temperature to 900 °C. The samples were held at 900 °C for 8, 48, and 96 h and then cooled inside the furnace.

The cross-sectional microstructural morphology of the corroded specimens was analyzed using scanning electron microscopy (SEM) with an F50 Inspect SEM equipped with an energy-dispersive X-ray spectrometer (EDS) EDAX APEX 2i and SDD Apollo X detector (FEI Company, Hillsboro, OR, USA). The acquired data were processed and analyzed using EDAX Genesis software v6.29 (EDAX Inc. Ametek MAD, Pleasanton, CA, USA). Backscattered electron images were acquired using a low-voltage high-contrast vCD detector (FEI Company) to enhance the contrast and distinguish the chemical composition variations at the corrosive layer–substrate interface. Microcompositional analyses were performed on two selected microareas using SEM-EDS based on the cross-sectional images of the specimens. The analysis was performed with a spot size of 3.5 nm and a working distance of 11.6–11.7 mm.

## 3. Results

The hot corrosion behavior of the investigated nickel-based superalloys (Inconel 718, Udimet 710, Nimonic 75, and Inconel 625) was assessed based on weight gain measurements after exposure to Na_2_SO_4_–V_2_O_5_ molten salt environments at 900 °C for 8, 48, and 96 h. The recorded weight gain values, which reflect the extent of oxide scale formation and corrosion product accumulation, are presented in Table 2.

**Figure 2:**
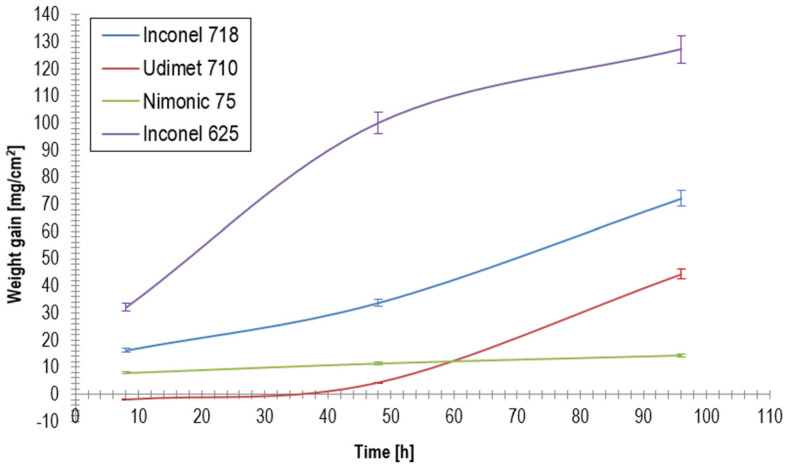
Weight gain evolution during hot corrosion testing.

To further illustrate the hot corrosion behavior of the investigated nickel-based superalloys, the weight gain (mg/cm^2^) data presented in Table 2 were plotted as a function of exposure time (8, 48, and 96 h), as shown in Figure 2.

Unusual behavior was observed for Udimet 710, which exhibited negative weight gain after 8 h, likely due to the volatilization of surface reaction products. While modest weight gain was observed at 48 h, a sharp increase was recorded at 96 h, suggesting a delayed breakdown of corrosion resistance. Although the precise mechanism remains unclear, this nonlinear progression may involve transient protective oxide formation, followed by rapid degradation owing to subsurface destabilization.

The differences in weight gain among the tested superalloys can be attributed to their chemical compositions. Nimonic 75 exhibited the best resistance of up to ~160% towards Inconel 625, whereas Udimet 710 initially lost material, but long-term exposure led to its degradation. Inconel 718 underwent progressive corrosion owing to Cr depletion and the formation of a secondary phase. Inconel 625 exhibited the worst performance, where a high Mo content may have facilitated the formation of low-melting-point compounds that accelerated corrosion.

**Figure 3:**
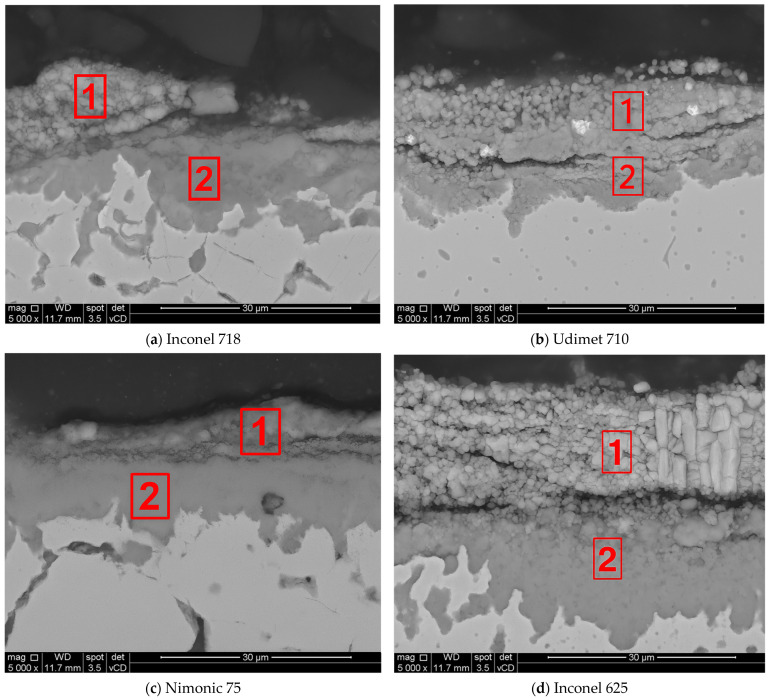
The micro-areas where SEM-EDS analysis was performed.

To further investigate the hot corrosion mechanisms in Na_2_SO_4_–V_2_O_5_ environments, scanning electron microscopy (SEM) with energy-dispersive X-ray spectroscopy (EDS) was performed on the samples of Inconel 625, Inconel 718, Nimonic 75, and Udimet 710 alloys. The SEM images in Figure 3 illustrate the corrosive scale morphology, corrosion products, and diffusion at the metal–oxide interface.

The regions marked as (1) correspond to the external corrosive layer formed by high-temperature corrosion product deposition. Region (2) corresponds to the internal corrosive zone, where elemental diffusion and secondary phase formation occur. Inconel 718 exhibited a multilayered structure, with region (1) displaying a porous and fragmented external scale. Region (2) exhibited internal corrosion, with a diffusion zone extending into the substrate. The cracks observed in the oxide scale suggest spallation and mechanical instability, which contribute to the increase in weight gain over time. The microstructure of Udimet 710 comprised a partially degraded external corrosive layer (1), characterized by localized porosity and cracks. The high Al (2.9 wt. %) and Ti (4 wt. %) content promotes the formation of γ′ (Ni_3_(Al,Ti)) phases, which improve corrosion resistance. However, the presence of W (1.5 wt. %) and Mo (2.89 wt. %) may contribute to the formation of volatile oxides, leading to localized material loss. Region (2) shows a well-defined corrosion front, suggesting a moderate degree of internal corrosion, but an overall better performance than that of Inconel 625 and Inconel 718. Nimonic 75, which exhibited the lowest weight gain, exhibited a compact and adherent corrosive scale (1) with limited porosity. The high Ni (74.9 wt. %) and Cr (20 wt. %) content in this alloy supports the formation of a stable and large protective layer, minimizing further degradation. Region (2) displayed a uniform internal oxidation zone with no significant evidence of secondary phase formation or Cr depletion. The superior oxidation resistance of Nimonic 75 can be attributed to the absence of Mo and Fe, which contribute to oxide instability. The microstructure of Inconel 625 after 96 h at 900 °C revealed a thick porous oxide layer (1), indicative of severe material degradation. A high Mo content (9.38 wt. %) in Inconel 625 likely contributed to the formation of Mo-rich compounds, which are known to be thermally unstable, accelerating corrosion. Beneath the corrosion layer, region (2) shows intergranular attack and subsurface corrosion, likely owing to the instability of the Cr_2_O_3_ scale under molten salt conditions. The high weight gain observed for Inconel 625 is consistent with this extensive degradation.

To further investigate the oxide scale composition, energy-dispersive X-ray spectroscopy (EDS) was conducted on specific microareas identified in the SEM images. Table 3 presents the chemical compositions (wt. %) of key elements, including O, Mo, Cr, Fe, Ni, Al, Ti, and Co, obtained from two selected areas in each specimen. These results provide insights into the corrosion layer stability, corrosion products, and elemental diffusion mechanisms of each tested alloy.

During this process, key protective corrosion products are formed, including spinels, such as NiCr_2_O_4_ and CoCr_2_O_4_, along with Cr_2_O_3_ and NiO [19,20].

By correlating the concentrations of Cr, Ni, Co, Al, Ti, and O in both zones 1 and 2 with Figure 2, it can be concluded that Cr, Ni, and Co play the most significant role in protecting the superalloy and neutralizing corrosive salts, followed by Al and Ti, which exhibit a relatively minor effect due to their lower concentrations in the superalloy.

## 4. Discussion

The results of this study provide a comprehensive understanding of the hot corrosion behavior of Inconel 718, Udimet 710, Nimonic 75, and Inconel 625 in Na_2_SO_4_–V_2_O_5_ environments at 900 °C, integrating weight gain measurements, SEM imaging, and SEM-EDS chemical analysis.

Weight gain analysis, shown in Figure 2, demonstrated that Inconel 625 exhibited the highest weight gain (127.14 mg/cm^2^ after 96 h), followed by Inconel 718 (72.07 mg/cm^2^), Udimet 710 (44.34 mg/cm^2^), and Nimonic 75 (14.33 mg/cm^2^), which exhibited the best hot corrosion resistance. The rapid degradation of Inconel 625 was attributed to its high Mo content (9.38%), which promoted the formation of thermally unstable Mo-rich compounds, leading to corrosion layer spallation and increased corrosion rates of the substrate. In contrast, Nimonic 75, with its high Ni (74.9%) and Cr (20%) contents, exhibited the lowest weight gain, indicating the formation of a stable protective scale that effectively inhibited further corrosion.

The SEM microstructural analysis shown in Figure 3 revealed significant differences in the corrosive scale morphology across the tested alloys. Inconel 625 exhibited a thick, porous, and unstable oxide layer, confirming its high susceptibility to corrosion. Inconel 718 exhibited a multilayered, fragmented corrosion scale, which contributed to an increasing corrosion rate over time. Udimet 710 exhibited a more compact but partially degraded corrosion layer with some porosity and cracks, indicating a moderate level of degradation. In contrast, Nimonic 75 exhibited a dense and adherent corrosive layer with limited internal corrosion, which is consistent with the superior resistance observed in the weight gain measurements.

SEM-EDS analysis, shown in Table 3, further supported these findings by identifying the elements’ distributions within the corrosive scale. Inconel 625 exhibited significant Mo enrichment (25.86%) in the corrosion layer, confirming the presence of Mo-rich compounds and leading to scale instability. Inconel 718 exhibited Cr-Ni oxides with localized Fe enrichment, suggesting selective corrosion and secondary phase formation, which likely contributed to the progressive weight gain of the alloy. In addition, Udimet 710 exhibited Mo accumulation (22.36%) in certain regions, indicating that its corrosion behavior was strongly influenced by Mo-rich compounds, although the presence of Al (2.69–3.73%) and Ti (2.51–3.18%) provided additional corrosion resistance through the formation of the γ′ phase. Nimonic 75 had the highest Cr and Ni content (42.91%/68.57%) in the layers, supporting the formation of a stable Cr_2_O_3_ protective scale and spinel phase NiCr_2_O_4_, which minimized further degradation.

The results of this study strongly indicate that Mo is a critical weak point in the hot corrosion mechanism of the investigated superalloys, particularly Inconel 625 and Udimet 710, in which Mo-rich compounds were identified by SEM-EDS analysis. The high Mo content in Inconel 625 (9.38%) led to the formation of thermally unstable Mo compounds, which are prone to volatilization in Na_2_SO_4_–V_2_O_5_ environments, accelerating the corrosion scale degradation and increasing the weight gain over time. Similarly, Udimet 710, which contains 2.89% Mo, exhibits localized Mo enrichment in the corrosion layer, further suggesting that Mo promotes the formation of porous and weakly adherent corrosive scales, reducing long-term corrosion resistance. Numerous studies have consistently confirmed that Mo-rich compounds are thermally unstable in molten salt environments, accelerating corrosion and oxide scale degradation [21,22,23,24].

The results also demonstrate that iron (Fe) negatively impacts the corrosion resistance of Inconel 718. The high Fe content (23.2%) in this alloy, coupled with the observed Fe-rich oxide formations in the SEM-EDS analysis, suggests that Fe oxidation contributes to porous and unstable corrosion layers, reducing the effectiveness of Cr_2_O_3_ as a protective barrier. This explains why Inconel 718 exhibited progressive weight gain, because Fe-rich oxides are less protective and more susceptible to spallation than Cr_2_O_3_. This behavior has also been reported in other studies [25,26].

Conversely, Al, Ti, and Co play complex roles in hot corrosion resistance. In Udimet 710, Al (2.9%) and Ti (4%) contributed to the formation of γ′ (Ni_3_(Al,Ti)) strengthening phases, which are known to improve corrosion resistance; however, their protective effect appears to be limited in molten salt environments. SEM-EDS analysis revealed that Al, Ti, and Co were present in the corrosion layers, suggesting that they contributed to protective oxide formation but were not as effective as Cr_2_O_3_ and spinel phase NiCr_2_O_4_ under high-temperature corrosive conditions. Additionally, the presence of Co (5.8–8.69%) in Udimet 710 suggests a potential role in enhancing corrosion resistance; however, its effects appear to be secondary to those of Cr and Ni.

The superior corrosion resistance of Nimonic 75 is attributed to its high Ni (74.9%) and Cr (20%) contents, as confirmed by the SEM-EDS analysis, which showed the highest Cr_2_O_3_ concentration in the corrosion scale (42.91 wt. %). Cr is well known to form dense and stable protective scales, preventing further corrosion and sulfidation, whereas nickel (Ni) stabilizes the alloy matrix and reduces Fe oxidation susceptibility. The minimal presence of Mo and Fe in Nimonic 75 further reinforces its excellent hot corrosion resistance, as it lacks the destabilizing effects observed in Inconel 625 and 718.

Although direct phase analysis was not performed, the significant Mo enrichment observed in the outer oxide layers of the corroded samples, particularly in Inconel 625, strongly suggests the formation of MoO_3_ (molybdenum trioxide). MoO_3_ is a volatile oxide with a relatively low melting point, and its presence is associated with the destabilization of protective Cr_2_O_3_ scales because of its tendency to evaporate or react with other constituents in the molten salt mixture. Moreover, it is plausible that low-melting-point eutectic phases may form between Mo oxides and other salt components (e.g., V_2_O_5_ or Na_2_SO_4_), further facilitating oxide scale fluxing, spallation, and internal oxidation. It can also be mentioned that in our previous study [27] using XRD analysis, the NiMoO_4_ compound was identified, which highlights the versatility of Mo oxide through its interaction even with Ni in a mass percentage of over 30% of the total analyzed powder, highlighting the possibility of reducing the protective spinel phase of Cr_2_O_3_ and NiO by reducing the amount of NiO in the layer. These mechanisms are consistent with the observed increase in weight gain and corrosion product porosity in Mo-rich alloys and support the conclusion that Mo acts as a destabilizing element in high-temperature molten salt corrosion reactions.

## 5. Conclusions

The results of this study confirm that the corrosion resistance of the studied superalloys (Inconel 718, Udimet 710, Nimonic 75, and Inconel 625) is strongly influenced by their chemical composition, particularly the stability of NiCr_2_O_4_, CoCr_2_O_4_, and Cr_2_O_3_, and the detrimental effects of Fe- and Mo-rich compounds. Nimonic 75 was the most resistant, whereas Inconel 625 was the most vulnerable, reinforcing the role of Cr-rich protective layers in mitigating high-temperature molten salt corrosion.

Mo and Fe contribute to scale instability and corrosion progression, whereas Cr and Ni significantly enhance resistance, as demonstrated by the superior performance of Nimonic 75. The findings suggest that using superalloys with lower Mo and Fe contents and higher Cr and Ni concentrations can significantly enhance the durability of superalloys in molten salt environments.

For future studies, a more complex, commercially available superalloy with no or minimal Mo and Fe, such as CMSX-4, CMSX-8, TMS-series, or René N6, should be used. It is also desirable to study the influence of Ta and Re elements in the hot corrosion process.

## Figures and Tables

**Table 1:** Chemical composition of raw materials (wt. %).

Elements	Ni	Cr	Mo	Fe	Nb	Co	Mn	Al	Ti	Si	C	W
Inconel 718	49.8	17	2.83	23.2	4.96	0.385	0.0799	0.582	0.765	0.0055	<0.005	<0.025
Udimet 710	55.8	18.2	2.89	0.257	<0.005	13.6	0.0295	2.9	4	0.546	0.061	1.5
Nimonic 75	74.9	20	0.0197	3.67	0.0518	0.0455	0.393	0.206	0.278	0.103	0.241	<0.025
Inconel 625	61.5	21.4	9.38	3.25	3.62	0.0794	0.0992	0.149	0.258	0.0425	0.0263	<0.0250

**Table 2:** Weight gain of specimens after hot corrosion tests.

Sample	Weight Gain [mg/cm^2^]		
	900 °C/8 h	900 °C/48 h	900 °C/96 h
Inconel 718	16.18 ± 0.65 mg/cm^2^	33.72 ± 1.35 mg/cm^2^	72.07 ± 2.88 mg/cm^2^
Udimet 710	−2.04 ± 0.08 mg/cm^2^	4.25 ± 0.17 mg/cm^2^	44.34 ± 1.77 mg/cm^2^
Nimonic 75	7.85 ± 0.31 mg/cm^2^	11.35 ± 0.45 mg/cm^2^	14.33 ± 0.57 mg/cm^2^
Inconel 625	32.13 ± 1.29 mg/cm^2^	99.97 ± 4.00 mg/cm^2^	127.14 ± 5.09 mg/cm^2^

**Table 3:** Chemical compositions obtained by SEM-EDS in the specific areas from Figure 3.

Elements		O K	MoL	CrK	FeK	NiK	Al K	Ti K	CO K
Inconel 718	Area 1	20.39	6.63	28.68	14.95	25.13	-	-	-
	Area 2	22.69	9.54	28.18	13.58	20.26	-	-	-
Udimet 710	Area 1	15.68	2.63	7.78	-	54.07	3.73	2.51	8.69
	Area 2	17.79	22.36	9.89	-	25.12	2.69	3.18	5.8
Nimonic 75	Area 1	23.21	-	42.91	3.84	27.53	-	-	-
	Area 2	16.25	-	12.24	1.47	68.57	-	-	-
Inconel 625	Area 1	19.34	25.86	9.47	2.18	24.5	-	-	-
	Area 2	26.1	6.85	29.93	4.15	23.23	-	-	-

## Data Availability

The original contributions presented in this study are included in the article. Further inquiries can be directed to the corresponding author.
